# Establishment of Vero cell-adapted thermostable candidate Newcastle Disease vaccine strain

**DOI:** 10.5455/javar.2025.l959

**Published:** 2025-09-22

**Authors:** Md. Humayun Kabir, Md. Amirul Hasan, Yu Miyaoka, Makiko Yamaguchi, Chisaki Kadota, Dany Shoham, Harumi Murakami, Kazuaki Takehara

**Affiliations:** 1 Laboratory of Animal Health, Cooperative Division of Veterinary Sciences, Graduate School of Agriculture, Tokyo University of Agriculture and Technology, Fuchu-shi, Japan; 2 Bangladesh Livestock Research Institute, Savar, Bangladesh; 3 Laboratory of Animal Health, Department of Veterinary Medicine, Faculty of Agriculture, Tokyo University of Agriculture and Technology, Fuchu-shi, Japan; 4 Begin-Sadat Center for Strategic Studies, Bar-Ilan University, Ramat Gan, Israel

**Keywords:** Antibody, Newcastle disease virus, thermostability, vaccine, Vero cell

## Abstract

**Objective:**

The current study aims to determine a thermostable Avian Orthoavulavirus 1 (AOaV-1) or Newcastle disease (ND) vaccine candidate strain isolated from a duck and its adaptation to Vero cells.

**Material and Methods:**

From field isolates of AOaV-1 in Japan, avirulent APMV/northern pintail/Japan/Aomori/2003 (dk-Amori/03, AOaV-1-261) was selected for its excellent thermostability, and the strain was heat treated at 56°C for 30 min during each passage into Vero cells to maintain thermostability and to adapt to Vero cells up to 20 passages, then named AOaV-1-Vero20. Growth curves were tested in Vero cells in comparison to the original AOaV-1-261. The intracerebral pathogenicity index (ICPI) test was done. The thermostability of the virus was checked at different temperatures. Serum neutralization test was done to check the induced levels of antibodies of the candidate vaccine strain in chicks. Sequencing of the *HN* and *F* genes and complete genome sequencing of the strains were done.

**Results:**

AOaV-1-Vero20 grew well on Vero cells compared to the original AOaV-1-261. The ICPI test score was 0. Minimum titer drops were found in the thermostability test. Induced, adequate levels of antibodies were measured using the serum neutralization test. Sequencing of the *HN* gene also revealed transmembrane domains, including 315 and 369 amino acid residues, with the same amino acids as those found in other thermostable strains. The sequence analysis results showed no significant variance in the whole genome sequences of AOaV-1-Vero20 and the original AOaV-1-261.

**Conclusion:**

In this study, AOaV-1-Vero20 was identified as a candidate vaccine strain that is thermostable, Vero cell-adapted, and has immunogenic potential.

## Introduction

Newcastle disease (ND) is a highly contagious and devastating disease that causes significant economic losses in the poultry industry by generating digestive, neurological, and respiratory symptoms [1]. In accordance with the seriousness of its symptoms occurring in chickens, Newcastle disease virus (NDV) has been categorized into three pathotypes: lentogenic, mesogenic, and velogenic. Low-virulent or avirulent NDV strains are known to cause subclinical infections with minor respiratory or stomach disorders. In contrast to velogenic NDV strains, which can cause mortality rates as high as 100%, mesogenic NDV strains are of intermediate virulence and cause respiratory infections with moderate mortality (<10%) [2]. Velogenic strains are further divided into two categories: neurotropic velogenic strains result in neurological and respiratory illnesses, while viscerotropic velogenic strains generate fatal hemorrhagic lesions in the digestive tract [3].

Low virulence strains derived from waterfowl, which cause no apparent symptoms of disease, are often utilized as candidates for potential live vaccines. Generally, live vaccines are heat sensitive and therefore require cold-chain conditions to maintain their effectiveness during storage and transport. For maintaining the vaccines at a low temperature, the cold chain may consume up to ~80% of the total expenses of vaccination programs [4]. In addition, the cold chain is not always reliable. Temperature excursions outside the recommended temperature range frequently occur during storage and transport due to inappropriate cold chain equipment, power shortages, and human error [5]. The challenges are even worse, especially in developing and underdeveloped countries. *HN* protein has been identified to be a crucial determinant of NDV thermostability [6].

ND vaccines are produced traditionally by propagating virus strains in embryonated chicken eggs. NDV is collected from the allantoic fluid and then processed into a vaccine. However, this traditional method poses some drawbacks, such as poor-quality control, high labor requirements, being a time-consuming process, and a high number of specific pathogen-free (SPF) eggs, which are the cause of the spread of some diseases and require a large area for the incubation of eggs [7].

Several reports have indicated that vaccines cannot provide complete protection in chickens even after ND vaccinations due to the new NDV variants with genetic distances [8]. However, it has also been reported that currently available avirulent vaccines can protect chickens from these emerging variants [9]. In Afghanistan, virulent NDV strains were detected in vaccinated chicken farms [10], and problems with proper vaccine storage were suggested.

For the particular difficulties of vaccinating multi-age flocks with relatively small numbers of chickens in areas with inadequate or inconsistent vaccine storage conditions, ND control in backyard flocks has been a struggle for decades [11]. ND vaccines with natural thermotolerance, artificial selection for increased thermotolerance, vaccine delivery food, and the use of chemicals for delaying thermal inactivation are some of the methods used over the past 30 years to try to control ND in village flocks better. Some commonly used thermostable vaccines are based on vaccine strains from class II genotype I (i.e., I2, V4, and PHY-LMV42). These strains are avirulent and can be safely administered via eye drops to chickens of all ages using freeze-dried forms [12]. Their increased stability to heat is particularly advantageous in remote areas of the world with limited refrigeration abilities. Although the thermostability of NDV may appreciably vary between strains, there is very little data on this attribute in reference to various virus strains. Previous studies have attempted to isolate avirulent, thermostable NDVs from the feces of wild migratory birds for vaccine candidates [13]. The current study aims to identify and establish an avirulent, thermostable, Vero-cell-adapted ND vaccine candidate strain isolated from a duck.

## Materials and Methods

### Ethical approval

Animal experimental work was performed strictly according to the Animal Care Guidelines of the Tokyo University of Agriculture and Technology (Tokyo, Japan), under permit number R03-28.

### Viruses and cells

NDV velogenic strain, namely Sato [5]; mesogenic strain, namely TCND; and lentogenic strains B1, Ishii, and APMV/northern pintail/Japan/Aomori/2003 (dk-Aomori/03, thereafter referred to as AOaV-1-261) were used [14]. Strains Sato, Ishii, and TCND were kindly supplied by the Kitasato Institute (Tokyo, Japan), and the vaccine strain B1 was purchased from Nisseiken Co., Ltd. (Tokyo, Japan).

Vero E6 cells (1.5 × 10⁵ cells/ml), kindly provided by the National Institute of Infectious Diseases (Musashimurayama, Tokyo, Japan), were cultured and passaged approximately 20 times. Primary chicken kidney (CK) cells were prepared in the Laboratory of Animal Health (Virology), Cooperative Division of Veterinary Sciences, Graduate School of Agriculture, Tokyo University of Agriculture and Technology, Japan, from SPF chickens, following previously described methods [15]. The culture media were the same as those used in our earlier study [16].

### Thermostable strain screening

Vials containing 0.5 ml aliquots of NDV strains, namely Sato, TCND, B1, Ishii, and AOaV-1-261, were retrieved from a -80°C deep freezer, and the aliquots were thawed. The samples were kept on ice or in a water bath at 56°C for 30, 60, and 120 min and then immediately transferred onto ice to halt the inactivating heat. All aliquots were determined for *HA* activity by standard methods [17]. The lentogenic virus, AOaV-1-261, retained sufficient *HA* activity after incubation at 56°C for 2 h and was selected for subsequent studies (Table 1).

### Thermostable AOaV-1 strain adaptation to the Vero cell line

At the time of each passage in Vero cells, AOaV-1-261 was heat-treated at 56°C for 30 min, inoculated onto Vero cells, incubated at 37°C for 60 min, and then supported with a maintenance medium (MM) containing 1 µg/ml trypsin from bovine pancreas (Sigma Chemicals, Saint Louis, MO, USA). At last, the virus was serially passaged up to 20 times in Vero cells, and the viruses at each passage were designated as AOaV-1-Vero1 to AOaV-1-Vero20.

**Table 1.** Effect of heat treatment (56°C) on hemagglutination activity of the AOaV-1-Vero20 after each passage on Vero cells from the original AOaV-1-261.

AOaV-1 strain	Time (min)	HA titer (log2)
AOaV-1-261	Before heat treatment	2^7^
AOaV-1 Sato	120	<2^2^
AOaV-1 TCND	120	<2^2^
AOaV-1 B1	120	<2^2^
AOaV-1 Ishii	120	<2^2^
AOaV-1-261	120	2^6^
AOaV-1-Vero1	30	2^5^
AOaV-1-Vero2	30	2^6^
AOaV-1-Vero3 to AOaV-1-Vero19	30	2^4 ^to 2^6^
AOaV-1-Vero20	30	2^7^

### Growth curve of the virus in Vero cells 

To determine the adaptation of the vaccine candidate to Vero cells, the replication kinetics of the original AOaV-1-261 and AOaV-1-Vero20 were compared. A monolayer of Vero cells grown in 6-well plates was inoculated with the viruses at the multiplicity of infection MOI of 0.01 plaque-forming unit (PFU)/cell. The Vero cell monolayer was washed thrice with phosphate-buffered saline (PBS) (pH 7.2), supplemented with the trypsin-containing MM, thereby leading to 1 µg/ml as the final concentration, and incubated at 37°C. The culture supernatants were collected at 12, 24, 36, 48, 72, and 96 h post-inoculation, centrifuged, and stored at 80°C until evaluated. These samples were titrated on Vero or CK cells for PFU/ml or a fifty percent tissue culture infectious dose (TCID_50_/ml) [18].

### Thermal stability test of the candidate vaccine strain

The candidate vaccine strain AOaV-1-Vero20 was evaluated for thermal stability at 37°C, 25°C, and 4°C storage temperatures for 0.5, 1, 1.5, 2, 5, 7, and 10 days. At every time point, the virus was harvested and titrated.

### Reverse-transcription polymerase chain reaction (RT-PCR) and sequencing of F and HN genes

RT-PCR and sequencing reactions were performed, and the sequence data were deposited in DDBJ, as described [9]. Specific primer sets were used to amplify the *F* gene [19] and *HN* gene of AOaV-1-261 and AOaV-1-Vero20 [20].

For the analysis, the first quarter of the coding region of the *F* gene (nucleotides 47 to 420), which includes essential structures such as the cleavage site, as well as the coding region of the *HN* gene (nucleotides 45 to 1685), was selected. Sequence analysis was performed using MEGA X software, version 10.1.8. Additionally, BLASTN searches together with reference strains in GenBank (http://blast.ncbi.nlm.nih.gov/Blast.cgi).

To evaluate the *HN* relationship between experimental strains and vaccine strains, multiple sequence alignment of the *HN* gene of AOaV-1-261 and AOaV-1-Vero20 was performed alongside other representative thermostable vaccines. The parameters used for the sequence analysis were multiple alignments (ClustalW), sequence identity plotter, and sequence matrix at both the amino acid and nucleotide levels.

### The intracerebral pathogenicity (ICPI) test

To test the level of pathogenicity of the candidate vaccine isolate by observing clinical signs and determining the mean death time in 1-day-old chicks over eight days [15].

### The immunogenicity of AOaV-1-Vero20 to chicks

The immunogenicity of AOaV-1-Vero20 toward chicks was evaluated after inoculating the virus into chicks. Experimental work on animals was performed in strict accordance with animal care guidelines of the Tokyo University of Agriculture and Technology (Tokyo, Japan) with permit number (R03-28). Six 1-day-old unvaccinated commercial layer chicks, hereafter referred to as “conventional chicks,” were purchased from Tomaru Co., Ltd. (Gunma, Japan), labeled, settled in plastic cages inside the isolators (CL-5443, Clea Japan, Tokyo, Japan), and kept until 4 days old, then used for the experiments. 

Blood samples were collected from chicks at 4, 14, and 30 days old to assess maternal antibodies. At 30 days old, the treatment group was inoculated with the virus of 10^6^ PFU per chick through an eye drop of 150 µl/bird. The control group received 150 µl PBS/bird. All the experimental birds were bled through a jugular vein at 37, 44, 51, and 60 days old, namely at 7, 14, 21, and 30 days post-vaccination (dpv).

Oropharyngeal swabs were collected from all chicks using a rayon cotton bulb swab (Japan Becton Dickinson, Tokyo, Japan) at 0, 2, 3, 5, 7, 14, and 21 dpv for isolation of vaccine viruses.

Virus neutralization (VN) test was done in CK cells to evaluate the chicken’s maternal and immune antibody efficacy toward NDV using the 50% plaque reduction method, with a constant amount of NDV (Sato strain) [5]. The neutralizing antibody titer at the 50% plaque-reduction point was assessed using Behrens-Karber’s method [21].

### Purification of the AOaV-1-26 and AOaV-1-Vero20

Both types of the viruses (before and after the thermostability and 20 passages for Vero cell adaptability) were kept in a −80°C deep freezer. Before purification, the viruses were cultured in CK cells and Vero E6 cells for AOaV-1-261 and AOaV-1-Vero20, respectively, in 225 cm^2^ flasks (2 flasks for each cell) with a dilution of 10 times with PBS. In this study, a centrifuge machine (BECKMAN COULTER^®^; made in the USA) was used for the purification of viruses.

### Next-generation sequencing and phylogenetic analysis

Whole genome sequencing against RNA virus using next-generation sequencing was carried out by Azenta Japan Corp. (formerly, GENEWIZ Japan). Briefly, the virus-derived RNA was quantified and qualified by Qubit RNA Assay (Thermo Fisher) and TapeStation RNA ScreenTape (Agilent). 25 ng of total RNA was used for cDNA library preparation without prior RNA selection/enrichment. The cDNA synthesis, followed by transcriptome library preparation, was conducted by the MGIEasy RNA Directional Library Prep Kit V2.0 (MGI tech), where dUTP was incorporated in the process of the 2nd strand cDNA synthesis instead of dTTP, which blocks PCR amplification against the 2nd strand templates, enabling strand-specific transcriptome profiling. A 13-cycle PCR amplification was performed to increase library yield. Resulting transcriptome sequencing libraries were quantified by Qubit DNA Assay (Thermo Fisher), and their fragment size distribution was confirmed by TapeStation D1000 ScreenTape (Agilent). The adapter sequences used in the library preparation are- forward: 5’-AAG TCG GAG GCC AAG CGG TCT TAG GAA GAC AA-3’, and reverse: 5’-AAG TCG GAT CGT AGC CAT GTC GTT CTG TGA GCC AAG GAG TTG-3’.

The resulting double-stranded library fragments were pooled/multiplexed at an equimolar amount each and further processed into single-stranded circular DNA (sscDNA), which is the final form of the MGI library. The sscDNA libraries were quantified by the Qubit ssDNA Assay Kit (Thermo Fisher) and used for generating DNA nanoballs (DNBs) by rolling circle replication reaction. DNBs were then loaded into a flow cell for sequencing on the DNBSEQ-G400 platform (MGI tech) with a 150 bp paired-end configuration, according to the manufacturer’s instructions. Essentially, 6.5 M paired-end reads were obtained for each sample.

### Statistical analysis

One-way analysis of variance (ANOVA), Student’s *t*-tests, and *F*-tests were performed for comparison of samples. *p*-values <0.05 were considered to be statistically significant.

## Results

### Thermostability

The AOaV-1-261 strain survived with an HA titer (64 log2) after exposure at 56°C for 120 min initially, whereas NDV Sato, NDV TCND, NDV Ishii, and NDV B1 showed < 4 log2. AOaV-1-Vero20 was exposed at 56°C for 30 min more than 20 times, and AOaV-1-Vero20 showed HA activity at 128 log2 HA titer (Table 1).

Virus titers (log_10_TCID_50_/ml) on Vero cells of AOaV-1-Vero10, AOaV-1-Vero15, and AOaV-1-Vero20 after heat treatment at 56°C for 30 min were 5.25 ± 0.14, 6.83 ± 0.08, and 8.00 ± 0.00, respectively.

### Virus growth curve in Vero cells 

**Figure 1.**
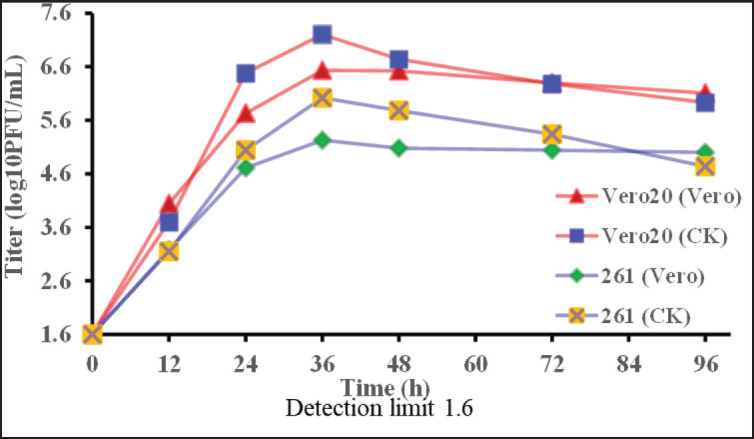
Graphical representation of growth kinetics of AOaV-1-261 and AOaV-1-Vero20 in the Vero cell line and CK cells (triplicate data, *t*-test, comparison between control and treatments, *p*-value < 0.05).

Multicycle growth kinetics of AOaV-1-261 and AOaV-1-Vero20 viruses in Vero cells at MOI 0.01 PFU/cell were titrated on Vero and CK cells (Fig. 1). AOaV-1-Vero 20 grew faster and higher in Vero cells than the original virus, AOaV-1-261, and the virus titers measured on CK cells were higher than on Vero cells (Fig. 1). The peak titers were measured at 36 h post-inoculation.

### Thermal stability test

**Figure 2.**
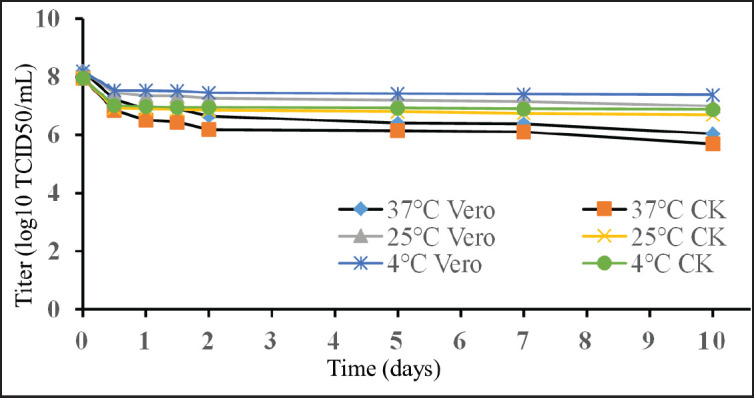
Infectious titer drops (log_10_ TCID_50_/ml) of the AOaV-1-Vero20 vaccine candidate, as maintained at 37°C, 25°C, and 4°C on CK and Vero cells (triplicate data, *t*-test, comparison between control and treatments *p*-value < 0.05).

The thermostability of AOaV-1-Vero20 was evaluated after incubation at 37°C, 25°C, and 4°C for 0.5, 1, 1.5, 2, 5, 7, and 10 days, respectively. The infectious titer (log_10_ TCID_50_/ml) slightly decreases over time at all selected temperatures, as shown in Figure 2.

### F and HN gene sequence analysis

The accession numbers of the sequences of part of the *F* gene and the complete *HN* gene for AOaV-1-261 and AOaV-1-Vero20 were LC709183, LC709184, LC709182, and LC709181, respectively. The same amino acid sequence was found in the *F* gene cleavage site of both AOaV-1-261 and AOaV-1-Vero20. These strains possess ^112^GKQGR*L^117^ at the cleavage site, which is an avirulent type.

**Figure 3.**
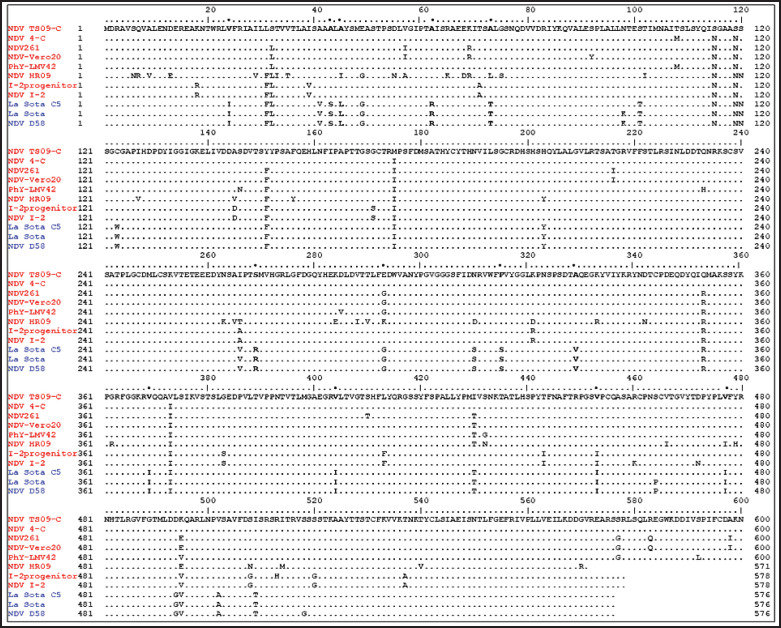
Multiple amino acid sequence alignment of HN proteins. Red and blue colors indicate thermostable and thermolabile strains. Black bold fonts marked by asterisks (*) denote point variation in identity between thermostable and thermolabile viruses.

The *HN* gene of both AOaV-1-261 and AOaV-1-Vero20 strains contains a coding sequence (CDS) comprising 1850 nucleotides coding for 616 amino acids. The amino acids at positions 315P and 369V in the *HN* gene were found, which have a major role in thermostability and *HA* and NA activity (Fig. 3). The transmembrane domains at positions 23–44 of both AOaV-1-261 and AOaV-1-Vero20 are like two predictable thermostable domains of the representative vaccine strains but differ from thermolabile strains, and point variations between thermostable and thermolabile viruses were shown (Table 2). The genome organization and the predicted protein length of the sequenced AOaV-1-261 and AOaV-1-Vero20 isolates were shown (Table 3). The sequence identity matrix for the *HN* gene of AOaV-1-Vero20, AOaV-1-261, and other representative thermostable and thermolabile vaccine strains of NDV in different genotypes (genotypes I, II, and VIII) was checked (Table 4). Thermostability was present in the AOaV-1-Vero20 strain irrespective of genotyping, which may be due to some similarities with their *HN* genes, which are responsible for thermostability.

Whole genome sequencing against AOaV-1-Vero20 and AOaV-1-261 viruses using next-generation sequencing was performed for molecular evaluation after heat treatment and adaptation on Vero cells to confirm its genome organization, protein length, and phylogenetic analysis. The results showed no significant variance in the genome sequences of AOaV-1-Vero20 and the original AOaV-1-261. Especially, no sequence variance in *F* and *HN* genes responsible for virulence and thermostability, respectively (Data not shown). A phylogenetic tree of AOaV-1-Vero20 and AOaV-1-261 based on complete *F* gene nucleotide sequence data, together with class II different genotypes/sub-genotypes, was made (Fig. 5). The candidate vaccine strain AOaV-1-Vero20 and its original state, AOaV-1-261, were mostly closed, and one Chinese duck strain was mostly near them. The complete genome sequences of the NDV261_duck_2003 and NDV-Vero20_duck_2003 strains were deposited in the DNA Data Bank Japan (DDBJ) database with the following accession numbers: LC723705 and LC723706.

### Immunogenicity of the strain AOaV-1-Vero20

**Figure 4.**
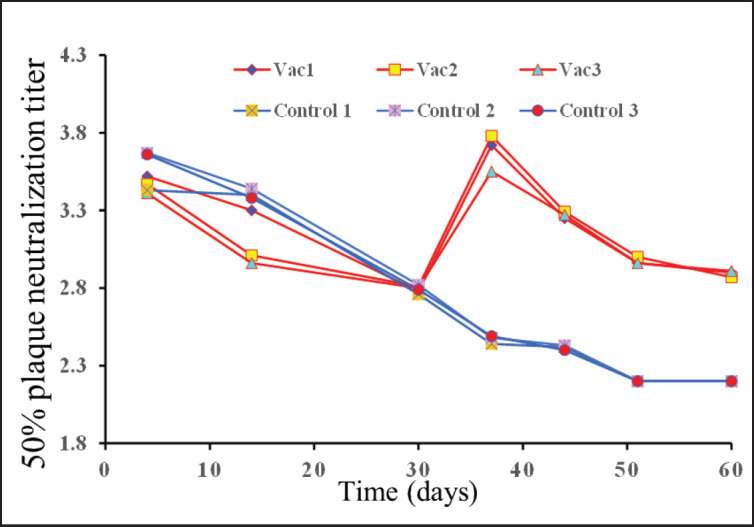
50% plaque reduction titer in log10 of NDV-specific antibodies of layer chickens following vaccination with AOaV-1-Vero20 candidate vaccine (triplicate data).

The virus shedding was confirmed by isolating AOaV-1-Vero20 in CK cells. The virus was recovered from all vaccinated chicks at 2, 3, and 5 dpv and 2 chicks at 7 dpv, but not from the mock-infected control. The result from the VN test showed that all chicks had high maternal antibody titers, which gradually decreased. After vaccination at 30 days old, the vaccinated group exhibited remarkably high titers and retained immunity up to 60 days old, whereas the control group’s passive immunity titer was diminishing to < 160 as shown (Fig. 4). An HI antibody titer of NDV 3 log2 (i.e., 1:8) and above is considered positive for specific immunity, and sera with antibody titers of 4 log2 have been reported. 

## Discussion

The effective prevention and control of ND usually depend on vaccination in many countries. In developing countries, biosecurity and immunization policies are not strictly applied. However, most live vaccines are sensitive to heat and subsequently require a cold chain to maintain the quality of vaccines during transport and storage. The cold chain may consume up to ~80% of the total cost of vaccination programs. Genotype I in class II, such as V4, Ulster, and others, commonly isolated from poultry, as well as some class I lentogenic viruses isolated from wild birds and live bird markets (LBM), are vaccine strain candidates, since they induce minimal adverse immune reactions [24-26]. For NDVs, thermostability is measured by HA activity or infectivity after exposure to a constant temperature (56°C) for different time intervals [27]. The criteria of the NDV thermostable lentogenic strains are their HA persistence longer than 30 min at 56°C and at least 20 min for a 2 log10 reduction of infectivity titer at 56°C temperatures [28]. The HA activity of thermostable strains remained at 56°C for 120 min, while other lentogenic thermolabile strains (B1, LaSota, and F strains) persisted only for 2 min [29]. Some thermostable strains, such as I2, HR-V4, Ulster, and V4, have been isolated, characterized, and found to furnish suitable protection efficacies [30].

In the present study, AOaV-1-261 was a confirmed thermostable lentogenic strain, and it was adapted to Vero cells. AOaV-1-Vero20 exhibited a high HA titer. This finding was supported by the findings for the thermostable NDV I-2 strain [23,30]. 

AOaV-1-Vero20 grew better than the original AOaV-1-261 in Vero cells (Fig. 1), and its high infectivity titer is similar to others’ findings [31,32], thus confirming its adaptation to Vero cells. 

In the thermal stability test, AOaV-1-Vero20 showed little decrease in infectious titer (log_10_ TCID_50_/ml) after incubation at 37°C, 25°C, and 4°C for 0.5, 1, 1.5, 2, 5, 7, and 10 days (Fig. 2). This finding is similar to the thermostable NDV I-2 strain [33].

The ICPI score of the strain was 0.0, indicating that it was an avirulent type [14]. The partial *F* gene sequence of both AOaV-1-261 and AOaV-1-Vero20 possesses ^112^GKQGR*L^117^ at their cleavage sites, which was denoted as an avirulent type.

The mutations of S315P and I369V in the HN protein could contribute to enhancing viral thermostability and HA and NA activity. These findings have important implications for understanding the thermostable mechanism of NDV and play a positive role in thermostable NDV vaccine development [22]. Thirteen amino acid residues at positions 123, 172, 186, 196, 238, 247, 251, 344, 455, 461, 465, 531, and 542 in *HN* protein have been reported to be essential for intramolecular disulfide bonds that stabilize the oligomeric *HN* structure gene, increasing the hydrophobic properties of the entire *HN* molecule responsible for thermostability after exposure to 56°C [34]; in that connection, findings found in the *HN* gene of AOaV-1-Vero20 are presented (Fig. 3).

**Table 2.** Predicted transmembrane amino acid sequences analysis of the *HN* gene of AOaV-1- AOaV-1-261, and commercially available thermostable and thermolabile vaccine strains.

AOaV-1 strain	Start	End	Length	Cutoff	Predicted amino acid sequence
AOaV-1-Vero20	23	44	22	1.7	LVFRIAILLLTVVTLAISAAAL
	25	42	18	2.2	FRIAILLLTVVTLAISAA
	208	222	15	1.7	LGVLRTSAIGRVFFS
AOaV-1-261	23	44	22	1.7	LVFRIAILLLTVVTLAISAAAL
	25	42	18	2.2	FRIAILLLTVVTLAISAA
	208	222	15	1.7	LGVLRTSAIGRVFFS
I-2^A^	23	44	22	1.7	LVFRIAILLLTVVTLAISAAAL
	25	42	18	2.2	FRIAILLLTVVTLAISAA
	424	430	7	1.7	ALLYPMI
	557	563	7	1.7	RIVPLLV
I-2 progenitor^A^	23	44	22	1.7	LVFRIAILLLTVVTLAISAAAL
	25	42	18	2.2	FRIAILLLTVVTLAISAA
	424	430	7	1.7	ALLYPMI
	557	563	7	1.7	RIVPLLV
TS09-C^A^	24	44	21	1.7	VFRIAILLSTVVTLAISAAAL
	25	42	18	2.2	FRIAILLSTVVTLAISAA
	210	211	2	1.7	VL
	424	430	7	1.7	ALLYPMI
	557	563	7	1.7	RIVPLLV
NDV4-C^A^	24	44	21	1.7	VFRIAILLSTVVTLAISAAAL
	25	42	18	2.2	FRIAILLSTVVTLAISAA
	210	210	1	1.7	VL
	424	430	7	1.7	ALLYPMI
	557	563	7	1.7	RIVPLLV
LaSota^B^	24	47	24	1.7	IFRIAILFLTVVTLAISVASLLYS
	25	45	21	2.2	FRIAILFLTVVTLAISVASLL
	557	563	7	1.7	RIVPLLV
LaSota C5^B^	24	47	24	1.7	IFRIAILFLTVVTLAISVASLLYS
	25	45	21	2.2	FRIAILFLTVVTLAISVASLL
	557	563	7	1.7	RIVPLLV
D58^B^	24	47	24	1.7	IFRIAILFLTVVTLAISVASLLYS
	25	45	21	2.2	FRIAILFLTVVTLAISVASLL
	557	563	7	1.7	RIVPLLV

The transmembrane domains marked with bold sequences showed similarities and variations between thermostable and thermolabile strains.^ A^Thermostable strains, ^B^Thermolabile strains.

The predicted amino acids present in the transmembrane domain of AOaV-1-Vero20 23LVFRIAILLLTVVTLAISAAL44 are similar to representative thermostable strains and different from thermolabile strains (Table 2) [23].

Our observed amino acid substitutions at V24I, A43S, A45L, E49G, A62R, A73T, N95K, S120N, C123W, H203Y, I266V, S269R, V369I, V404I, V453I, S464P, V477I, D494G, E495V, and I509T in our thermostable AOaV-1-Vero20 strain also support the explanation of thermostability (Fig. 3) [23]. Thermostability was present in the AOaV-1-Vero20 strain irrespective of genotyping, which may be due to some similarities with their *HN* genes, which are responsible for thermostability (Table 4) [34]. From whole genome sequencing of the AOaV-1-261 and AOaV-1-Vero20 strains, it was found that the genome organization and the predicted protein length of the sequenced AOaV-1-261 and AOaV-1-Vero20 were the same. There were no substitutions in the *HN* and *F* genes.

**Table 3.** Summary of the genome organization and the predicted protein length of the sequenced NDV261 and NDV-Vero20 isolates.

Gene	Gene start position (nt)	3’UTR length (nt)	Coding sequence positions (nt)	5’UTR length (nt)	Gene end position (nt)	Intergenic region length (nt)	Gene length (nt)	Protein length (aa)
*NP*	56–65	66	122–1591	210	1791–1801	2	1746	489
*P*	1804–1813	83	1887–3074	180	3245–3254	1	1451	395
*M*	3256–3265	34	3290–4384	112	4487–4496	1	1241	364
*F*	4498–4507	46	4544–6205	84	6280–6289	31	1792	553
*HN*	6321–6330	91	6412–8262	60	8313–8322	47	2002	616
*L*	8370–8379	11	8381–14,995	77	15,063–15,072	–	6703	2204

*F* = Fusion protein; *HN* = Hemagglutination-neuraminidase protein; *L*= Large polymerase enzyme; *M* = Matrix protein; *NP* = Nucleocapsid protein; *P* = Phosphoprotein;.

**Table 4.** Sequence identity matrix (%) for CDS of *HN* gene.

Sequence	^a^NDV Vero20	^a^NDV 261	^a^I-2	^a^I-2 progenitor	^a^TS09-C	^a^NDV4-C	^a^PHY-LMV42	^c^HR09	^b^LaSota	^b^LaSota C5	^a^D58	^a^Ishii
AOaV-1-Vero20	ID	99.82	95.59	95.77	97.43	97.98	98.34	92.29	94.30	94.67	94.12	98.63
AOaV-1-261	99.72	ID	95.40	95.59	97.24	97.79	98.16	92.29	94.12	94.49	93.93	98.90
I-2	91.36	91.26	ID	99.48	95.67	96.19	95.58	90.19	93.06	93.40	92.88	95.95
I-2 progenitor	91.36	91.26	99.84	ID	95.85	96.37	95.76	90.3	93.23	93.58	93.06	96.13
TS09-C	93.15	93.06	95.47	95.50	ID	99.03	97.28	90.89	93.75	94.10	93.58	97.79
NDV4-C	93.39	93.29	95.55	95.59	99.81	ID	98.16	91.42	94.27	94.62	94.10	98.34
PHY-LMV42	93.30	93.25	92.98	92.98	95.30	95.57	ID	91.59	94.29	94.66	94.11	98.46
HR09	86.74	86.68	85.52	85.52	86.05	86.16	85.93	ID	90.19	90.54	90.02	92.12
LaSota	88.43	88.34	87.74	87.77	89.51	89.61	89.02	84.12	ID	99.65	99.83	94.66
LaSota C5	88.57	88.48	87.83	87.83	89.61	89.72	89.24	84.18	99.82	ID	99.48	95.03
D58	89.15	89.09	88.48	88.48	89.58	89.81	89.72	84.06	99.94	99.83	ID	94.48
Ishii	94.49	94.43	94.08	94.08	96.19	96.42	97.27	86.75	90.30	90.36	90.35	ID

^a^Genotype I, ^b^Genotype II, ^c^Genotype VIII. Amino acid differences are indicated in bold, and nucleotide differences are in normal font. The difference from commonly used vaccine strains is highlighted.

When three 30-day-old chicks were inoculated with AOaV-1-Vero20, the virus was recovered until 7 days old, and the VN titer remained at protective levels for more than 30 days (Fig. 4). There was no weight loss observed in the vaccinated birds, and no clinical signs were observed in the vaccinated birds.

Our finding of the thermostability of AOaV-1-Vero20 was supported by the findings of Wen et al. [6], Ruan et al. [22], Omony et al. [23], Lomniczi B [28], and Bensink and Spradbrow [30], who identified thermostable NDV strains exhibiting persistence after heating at 56°C, and also on the molecular basis of the *HN* gene for thermostability. Therefore, from the findings of the present study, it is said that the experimental strain was the most robust candidate vaccine strain that can resist high temperatures and thus be advantageously used in rural areas and in tropical or subtropical countries, including Afghanistan and Bangladesh, especially for village flocks. Future research should focus on assessing large-scale field applications and other species like ducks, pigeons, and so on, to further validate their efficacy and sustainability in ND control programs.

**Figure 5.**
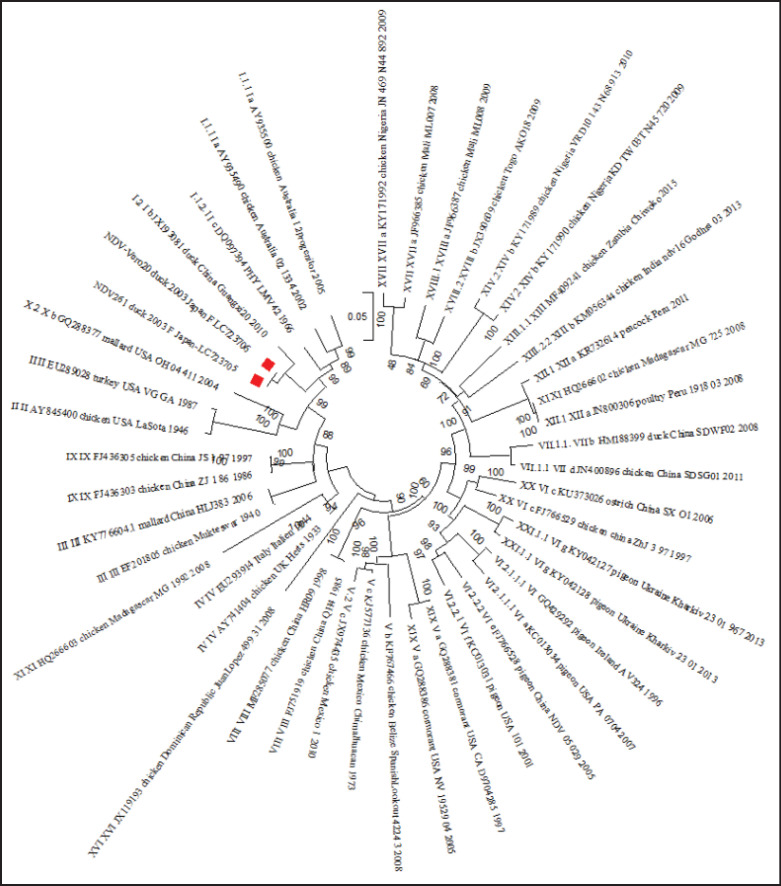
Phylogenetic tree of Newcastle disease virus based on complete F gene nucleotide sequence data of our isolates, together with class II different genotypes/sub-genotypes from the current classification system. The tree was constructed using the maximum likelihood method (1,000 bootstrap replicates) in MEGA-X software. Bootstrap values were shown at the nodes. The sequences determined in this study were marked with a square.

## Conclusion

Based on these outcomes of the present study, it was concluded that the AOaV-1-Vero20 strain is an avirulent, appropriately thermostable, Vero cell-adapted, highly immunogenic, and low cumulative titer drop candidate vaccine strain, which constitutes a proper alternative to traditional embryonated chicken egg-passaged-based vaccines. This thermostable vaccine candidate strain is expected to help control ND in Afghanistan and tropical countries, including Bangladesh.

## List of abbreviations

ANOVA, Analysis of variance; AOaV-1, Avian Orthoavulavirus 1; Bp, base pair; CPE, Cytopathic effect; cDNA, Complementary deoxyribonucleic acid; CK, Chicken kidney; °C, Degrees Celsius; dpv, Day post vaccination; DNA, Deoxyribonucleic acid; EMEM, Eagle’s minimum essential medium; FBS, Fetal bovine serum; GM, Growth medium; HA, Hemagglutination; h, hour; ICPI, Intracerebral pathogenicity index; min, minutes; ml, milliliter; MM, Maintenance medium; µl, microliter; MOI, Multiplicity of infection; ND, Newcastle disease; NDV, Newcastle disease virus; PBS, Phosphate-buffered saline; PFU, Plaque-forming unit; RT-PCR, Reverse transcriptase polymerase chain reaction; SPF, Specific pathogen-free; TCID_50_, Fifty percent tissue culture infective dose; VN, Virus neutralization.
